# A comprehensive postpartum follow-up health care program for women with history of preeclampsia: protocol for a mixed methods research

**DOI:** 10.1186/s12978-018-0521-8

**Published:** 2018-05-18

**Authors:** Mastaneh Kamravamanesh, Shahnaz Kohan, Negin Rezavand, Ziba Farajzadegan

**Affiliations:** 1 Student Research Center, Faculty of Nursing and Midwifery, Isfahan University of Medical Sciences, Isfahan, Iran; 2 Reproductive Health, Nursing and Midwifery Care Research Center, Faculty of Nursing and Midwifery, Isfahan University of Medical Sciences, Isfahan, Iran; 3 Obstetrics and Gynecology, School of Medicine, Kermanshah University of Medical sciences, Kermanshah, Iran; 4 Community medicine Department, Faculty of medicine, Isfahan University of Medical Sciences, Hezar Jereb St, Isfahan, Iran

**Keywords:** Preeclampsia, Postpartum care, Postpartum follow up, Needs, Mixed methods study, Intervention program, Health promotion, Lifestyle

## Abstract

**Background:**

Long-term postpartum follow-up is of great importance since women with preeclampsia history are at high risk of upcoming health complications. However, postpartum follow-up rates are poor. According to evidences, preeclampsia is not just a transient health problem; rather it causes short term and long term complications, which affect women’s life for years after delivery. Although it seems the problem is solved by the end of pregnancy, the follow-up of subjects should not be stopped after delivery. Postpartum is the best possible time to provide necessary care to these women who are at the risk of future complications. Due to importance of well-designed follow-up plan for women suffering preeclampsia, this study will carry out to provide a postpartum follow-up health care program for subjected women.

**Methods:**

This study is a qualitative-quantitative mixed sequencing exploratory study that consists of three consecutive phases. In this study, following a qualitative approach, the researcher will explain the needs and strategies related to promoting the health of women with preeclampsia history in the postpartum period. By entering the second phase, the researcher will design a comprehensive follow-up health care program in the postpartum period in which, in addition to using the qualitative study results, related papers and texts will be also used. The proposed program is designed by a panel of experts based on prioritization guidelines. Finally, after passing different stages of program finalizing, its effectiveness on the lifestyle of women with preeclampsia history will be investigated in a semi-experimental study in the third phase of the study.

**Discussion:**

It is expected conducting a mixed method study to design and execute an interventional program to follow up women with preeclampsia history improve their health status and well-being, while reducing their health care costs through prevention in various levels within the current structure of health care services. If this program is effective, it could be included in the postpartum health care guidelines.

**Trial registration:**

IRCT20170927036445N2 Registered 10 March 2018.

## Plain English summary

According to evidences, preeclampsia is not just a transient health problem; rather it causes short term and long term complications, which affect women’s life for years after delivery. Although it seems the problem is solved by the end of pregnancy, the follow-up of subjects should not be stopped after delivery. The results of this study offer a rich source of information for the required interventions to promote the health of women with preeclampsia history. This study is a qualitative-quantitative mixed sequencing exploratory study that consists of three consecutive phases. In this study, following a qualitative approach, the researcher will explain the needs and strategies related to promoting the health of women with preeclampsia history in the postpartum period. By entering the second phase, the researcher will design a comprehensive follow-up health care program in the postpartum period in which, in addition to using the qualitative study results, related papers and texts will be also used. The proposed program is designed by a panel of experts based on prioritization guidelines. Finally, after passing different stages of program finalizing, its effectiveness on the lifestyle of women with preeclampsia history will be investigated in a semi-experimental study in the third phase of the study. Therefore it is expected conducting a mixed method study to design and execute an interventional program to follow up women with preeclampsia history improve their health status and well-being, while reducing their health care costs through prevention in various levels within the current structure of health care services.

## Background

Preeclampsia is known as a major consequence of pregnancy even fatal for both mothers and infants throughout the world [1–6]. Although the clinical symptoms of preeclampsia are usually resolved after the delivery, it’s clinical and biological complications may continue up to several weeks or even months, causing health problems such as decreased insulin sensitivity, hypertension and endothelial dysfunction known as metabolic syndrome at long time after delivery [7–12]. Studies on the long term complications of preeclampsia in affected patients show that hypertension, ischemic heart disease, stroke, venous thromboembolism, renal failure, chronic kidney disease and even mortality rate are more common than general population. Early preeclampsia has the highest risk of fatal ischemic heart disease, as the pathophysiology of preeclampsia and cardiovascular disease are common [3, 6, 8, 13–23]. As a result, American Heart Association (AHA) considers a previous history of preeclampsia as a risk factor for cardiovascular disease [5, 12, 13, 17].

A history of preeclampsia of early onset can probably affect many aspects of women’s health including their fertility. It may also affect their decision for a next pregnancy [24]. Furthermore, other studies have shown that severe cases of preeclampsia and HELLP (Hemolysis, Elevated Liver enzyme, Low Platelet) syndrome caused women to seek psychological support in the years following delivery and some of them avoided next pregnancies due to their fear of HELLP syndrome [24–26]. Preeclampsia can lead to psychological and cognitive pathology related to trauma, such as post-traumatic stress syndrome and depression, which ultimately affect cognitive performance [14, 17, 27].

According to existing evidence, preeclampsia is not just a transient health problem and can lead to short- and long-term complications and it affects women’s quality of life even years after giving birth [28]. Therefore, postpartum is the best motivating time to modify their lifestyles [29]. Women who have experienced preeclampsia will achieve more benefits by changing their healthy lifestyle, if a regular follow up program are available that addresses risk factors well [8, 24, 30]. AHA also suggests that women with a previous history of preeclampsia should modify their lifestyle to reduce the risk of cardiovascular diseases [13, 17]. Likewise, several international and Canadian organizations have advised postpartum routine follow-ups for women with a history of preeclampsia to provide a healthy life style oriented consultation services [9, 14, 18, 31].

As a routine, most women who experience preeclampsia are considered as “managed” cases after delivery, and return to primary care system without follow-up program to prevent cardiovascular diseases [11, 12, 30, 32, 33]. Schaaf et al. (2011) also have recommended further research to evaluate the importance of structured follow-up programs to address physical and mental health as well as fertility of patients [24]. Although it seems the problem was solved by the end of pregnancy, the follow-up of subjects should not be stopped after delivery [8, 14, 16, 18, 34].

Women with a history of preeclampsia should be referred for receiving postpartum follow-up care and a holistic care would be addressed. Given that, there is not a comprehensive and integrated postpartum follow-up care for these women in Iranian health system, so this study will carry out to provide a postpartum follow-up health care program for women with history of preeclampsia.

### Objectives

The objectives of each phase are as follows:

**Objectives of the first phase: qualitative study.**

Explaining the needs of postpartum care in women with preeclampsia history.

**Objectives of the second phase: program design.**

Designing a preliminary intervention program based on the needs extracted from the qualitative phase and the review of texts.

Validating the intervention program by a panel of experts.

**Objectives of the third phase: quantitative study.**

Determining the effect of comprehensive postpartum follow-up health care program on the lifestyle of women with preeclampsia history.

## Methods/design

This study is a qualitative-quantitative mixed sequencing exploratory study that consists of three consecutive phases. In this study, following a qualitative approach, the researcher will explain the needs and strategies related to promoting the health of women with preeclampsia history in the postpartum period, and will examine the perceptions and experiences of the participants for this reason. In this part of the study, qualitative data will be collected through deep, semi-structured interviews and taking notes in the field. Participants eligible for participation in the study will be selected in a purposive manner and with maximum diversity. Simultaneously with data collection, the interviews will be analyzed by the conventional content analysis method. The researcher will continue sampling and encoding data until reaching saturation. After reaching saturation and ending the interviews, the researcher will enter the second phase of the study and design a comprehensive follow-up health care program in the postpartum period, in which, in addition to using the qualitative study results, related papers and texts will be also used.

Then, this proposed program will be validated by a panel of experts based on prioritization guidelines. Finally, after passing different stages of program finalizing, its effectiveness on the lifestyle of women with preeclampsia history will be investigated in a semi-experimental study in the third phase of the study. The collected data will be entered into SPSS statistical software and the data will be analyzed by descriptive-analytical statistics.

### First phase: Qualitative study

The first phase of the present study was designed to answer the question “What are the needs for promoting the health of women with preeclampsia history in the postpartum period?” This study is carried out using qualitative content analysis method.

### The research community and participants in the first phase (qualitative):

In the qualitative part of the present study, the research community will be considered to be the women with preeclampsia history who referred to hospitals, the service providers who have experience in providing health care to women with preeclampsia history, and maternal health policy makers.

### Selection of participants in the first phase (qualitative):

In the present study, the selection of participants will be done by purposive manner and with maximum diversity. Women with preeclampsia history will be selected with maximum diversity in terms of severity of the disease, disease duration, age, number of pregnancies, education, social class, occupation and place of residence, and service providers who have experience in providing health care to women with preeclampsia history will also be selected.

### Women with preeclampsia history will be included in the study by the following criteria:

Having informed consent to participate in the research and being able to communicate and conduct interviews.

Being Iranian and able to understand and speak Persian.

Not having cancer or hard-to-treat diseases.

Not having various types of severe psychiatric disorders (psychosis, schizophrenia, etc.) that are under medical treatment or need hospitalization.

### The criteria for entry of health care providers into the study:

Having at least 6 months of experience in providing health care to women with preeclampsia history.

Having the desire to participate in the study.

### Research environment:

Interviews will be conducted in coordination and with the views of the participants at the time and place designated by them wherever they feel comfortable (hospital, health centers, work places, university, home, etc.).

### Data collection process in the first phase (qualitative)

After referring to the hospitals affiliated to Isfahan University of Medical Sciences, the researcher make necessary coordination and examination of the delivery files; then, appropriate participants are selected and contacted through the address and telephone number contained in the files for participating in the study. After introducing, explaining the purpose of the study and the method of doing the study, participants are assigned an appointment in a private and comfortable environment (based on the choice of the participants).

In the qualitative part of the present study, the main data collection method will be deep, open and semi-structured interviews and taking notes in the field.

The interviews will be semi-structured, individually done and will take place within 1–2 sessions. Interviews will be recorded with sound recorder. Before the interview begins, the researcher will be re-introduced, explain the purpose of the interview, and get a verbal and written consent from the participant to participate in the research, continue cooperation, conduct further interviews and allow the recording of the interview. In case of not desiring to record sound, the researcher first tries to persuade her to record sound by establishing a good relationship with the participant. In the next step, the participant’s concern will be investigated and if her concern is the principle of confidentiality, the researcher will try to make a commitment that her confidentiality is fully respected. Finally, if the participant insists on not recording sound, the interview will be done without recording, but taking notes will be done more quickly.

In the semi-structured individual interviewing method, the first several interviews are conducted to get acquainted with probable and unpredicted issues. Using the resulting information, general questions for the semi-structured interviews will be identified. After the end of each interview, the interview will be transcribed at the earliest opportunity and data analysis will be carried out simultaneously with data collection. Data collection will continue until the data saturation stage is reached, that is, until no new data code is extracted. Not extracting new data will confirm saturation and adequacy of data.

### Data analysis of the first phase (qualitative):

In the present study, the conventional content analysis method proposed by Graneheim & Lundman (2004) will be used for data analysis. In this study, each interview will be immediately written word by word after recording, and after extracting its general idea, the text will be read line by line, semantic units will be determined, and from these units, compressed semantic units and codes will be extracted. After extracting the initial code, data reduction will be done, and eventually categories, Subcategories and the main categories respectively will appear from these codes [35].

### Accuracy and reliability of qualitative data:

To ensure the accuracy of the study and the reliability of the results, four of the proposed benchmarks of Polit & Beck (2018): credibility, dependability, transferability and confirmability, will be used [36].

In the present study, in order to increase the validity of the study, participants will be selected with maximum diversity, sufficient time will be assigned for data collection, multisession deep interviews will be conducted at different times and locations, and the integration of multiple data collection methods, such as interviewing and taking notes in the field will be used. The method of reviewing by the participants will be used to verify the accuracy of the extracted data and codes or to modify them.

In order to validate the findings, the external observer’s method will be used to investigate their probable similar perception with that of the researcher, and the search will be used for contradictory cases, which will be done by presenting initial codes from the perception of the experiences of participants, examples of how to extract codes and excerpts from the interview text for each of the codes.

In order to increase the transferability, the research results will be presented to a number of people having the characteristics of participants and who will not participate in the research for them to judge on the similarity between the research findings and their experiences.

The researcher will help increasing the confirmability of the research by maintaining documents and expanded reports, as well as excerpts from the interviews, along with the codes and categories extracted to be reviewed and commented by researcher colleagues and a number of faculty members who are familiar with analyzing qualitative researches and will not participate in this research, and by asking them to examine the authenticity of the data encoding process.

### Phase II: Designing intervention program

After reaching the findings of the conducted interviews, the second phase of the study, designing an intervention program to promote the health of women with preeclampsia history in the postpartum period, will begin. In fact, at this phase, based on the findings of the qualitative phase of the study and the information obtained from the texts and papers, postpartum health promotion strategies of this group of women will be determined following the preeclampsia delivery and will be validated in the panel of experts. Texts reviewing will be done by the Narrative Review method, including searching in electronic resources to gain access to knowledge related to issue. The researcher will also use library resources (reviewing reference books and theses) to access further information. Several databases are used to search and identify related articles, including PubMed, ScienceDrect, Web of Science, Cochrane Library, Scopus, ProQuest, Ovid, Magiran, SID, MEDLINE ،Embase، CINAHL Google scholar. At this phase, all studies published between 2006 and 2018 both in English and Persian languages with qualitative, quantitative, and mixed design with contents including preeclampsia, its short-term and long-term complications, postpartum care, health of women with preeclampsia history, health promotion and lifestyle interventions of these women, as well as postpartum health follow up, are reviewed. In the next phases, the search is performed with keywords and with different combinations including Preeclampsia; Preeclampsia AND “Postpartum care” plus preeclampsia AND “postnatal care” plus preeclampsia and “postpartum complication” plus preeclampsia AND “postpartum follow up” plus preeclampsia AND “postpartum lifestyle” plus preeclampsia AND “women experience” plus preeclampsia AND “women perception” plus preeclampsia AND “postpartum health promotion”plus preeclampsia AND “postpartum health improvement” plus preeclampsia AND “Postpartum Risk management”.

### Holding a panel of experts

At this stage of the research, the intention is to prioritize the strategies extracted from the qualitative study and the review of texts in the panel of experts in terms of their applicability, and then an intervention program based on the prioritized strategies by the experts is designed and applied in the quantitative phase. In this way, the decision matrix will be used to prioritize the strategies extracted from the qualitative study and the review of texts. In this matrix, a score between 1 and 3 will be assigned to each proposed strategy based on three criteria: cost, ease of implementation and time. This matrix will be sent to a number of experts in the first Delphi round, the average score for each solution will be determined and the solutions with the highest priority will be determined. An intervention program will then be designed for the strategies with the highest priority. In the second Delphi round, the proposed intervention program will be reviewed and evaluated qualitatively at a meeting with the presence of the research team and panel members (including specialists in obstetrics and gynecology, reproductive health, nutrition, heart, internal medicine and psychology, psychic nurse,, midwives and maternal health policy-makers). A copy of the intervention program is also provided to these experts to include their comments into the program and return it to the researchers. Comments and suggestions collected in the program will be implemented, and the designed intervention program will be finalized and implemented in the third phase (quantitative study).

### Phase III: Quantitative study

#### Type and direction of the quantitative study

The quantitative phase of the research will be implemented as a two-group semi-experimental study.

### The studied population

Target population for study is all women with confirmed diagnosis of preeclampsia.

### Research sample

The research sample will be formed from a group of women with preeclampsia who entered the study with easy sampling and have entry requirements for the study.

### Research environment

This research will be carried out in postpartum wards of hospitals affiliated to Isfahan University of Medical Sciences and maternal and child health centers. The reason for choosing such environment is convenient approach to women with the characteristics of the research unit. Further, the number of referees to these centers with characteristics of the research units is sufficient and includes the majority of cultural and social classes.

### Sample size

With a confidence level of 95% and a statistical power of 90%, along with considering the standard deviation of lifestyle score, which is equal to P_1_ = 0.87 and P_2_ = 0.69 and with EFFECT SIZE of 0.18, the sample size is considered to be 43 in each group.

### Sampling method

The sampling of this part of research is carried out by available and non-probabilistic sampling method. Researcher by referring to postpartum wards of hospitals affiliated to Isfahan University of Medical Sciences on consecutive days selects 86 women with preeclampsia and having the criteria for entering the study in the easy method. Subsequently, subjects are assigned to the intervention and control groups by means of the RANDOM ALLOCATION software.

#### Entry criteria

Ages ranging from 15 to 49 years, Iranian citizenship, consent to participate in the study, ability to understand questions or have reading and writing skills, not participating in simultaneous clinical trials, not having various types of severe psychiatric disorders (psychosis, schizophrenia, etc.) who are under medical treatment or in need of admission, women with preeclampsia who are nulliparous and whose delivery has led to the birth of a live newborn.

#### Exclusion criteria

Failure to receive 50% of the intervention for any reason, having serious complications of preeclampsia, such as nephropathy and being under drug treatment, severe life stress such as spouse’s death and immigration.

### Study variables

In this study, the designed interventions will be considered as an independent variable, and lifestyle will be considered as a dependent variable.

### Data collection method

The lifestyle questionnaire, which is completed by the interview method, is the tool used in the quantitative phase of this research. The questionnaire includes 70 questions, which designed to evaluate various aspects of lifestyle (e.g. physical health, exercise, weight and nutrition control, disease prevention, psychological health, spiritual health, social health, avoidance of medicines and drugs, prevention of accidents and environmental health). The scoring scale is in Likert format. The response range is assessed based on frequency of action. The frequency of action is measured as “always, often, sometimes, very rare, rarely”. The total score for each aspect comes from the sum of scores.

### The implementation method of the quantitative phase

The researcher will implement the designed intervention after obtaining a permit from the Ethics Committee of the Isfahan University of Medical Sciences, and performing the necessary coordination with the officials of the specified centers. In this way, the researcher first discuss the research objectives by attending predetermined referral centers; then, through the available sampling method to the research units having criteria for entering the study, and, if they agree and give informed consent, the person enters in the study. Life style of participants will be considered as a proposed outcome.

### Data analysis

The collected data will be analyzed using descriptive statistical methods (mean, standard deviation and frequency) and inferential statistics (independent t, paired t-test, Chi-square, Fisher’s exact test, Wilcoxon and Mann-Whitney) and by using SPSS 20 software. If there are confounding variables, ANCOVA will be used to modify them.

## Discussion

Various studies suggest that women with preeclampsia history need serious follow-up to manage the risk factors of chronic and cardiovascular diseases [8–10, 17, 18, 37–41]. Shortage in awareness of maternal care providers and lack of comprehensive instructions are the main reasons for neglecting them [11, 33, 34, 42, 43]. Further, missing evidence of cost-efficient post-preeclamptic follow-up strategies along with possible differences in clinical recommendations converge to indicate the demand for further research to improve and optimize prevention approaches [5].

Researchers also support studies on applying postpartum lifestyle interventions on women who have preeclampsia history as a strategy to prevent future cardiovascular disease and promote their health [9, 33, 44].

Due to importance of well-designed follow-up plan for women suffering preeclampsia [31], the present study aims to design and conduct a comperhensive postpartum follow-up health care program for participants. The program will be designed in a framework to provide regular and structured follow-up to reduce postpartum morbidity and improve women’s health through lifestyle change by evaluating and controlling the risk factors, which can lead to improving quality of life and longer life by preventing chronic diseases, as well as reducing the health care costs. This type of integrated intervention can be considered a significant strategy to reduce risk costs effectively. As a result, it is expected that the success of providing such a program, to some extent, could prevent health problems among these women who might be neglected due to high burden of disease. If this program is effective, it could be included in the postpartum health care guidelines. The results of the study will be provided to maternal health policy-makers in the health system.

## Abbreviations

AHA: American Heart Association
HELLP: H: hemolysis EL: elevated liver enzyme LP: low platelet
